# Effects of a Formula with scGOS/lcFOS (9:1) and Glycomacropeptide (GMP) Supplementation on the Gut Microbiota of Very Preterm Infants

**DOI:** 10.3390/nu14091901

**Published:** 2022-05-01

**Authors:** Xue Yu, Yan Xing, Hui Liu, Yanmei Chang, Yanxia You, Yuqi Dou, Bin Liu, Qi Wang, Defu Ma, Lijun Chen, Xiaomei Tong

**Affiliations:** 1 School of Public Health, Peking University Health Science Center, Beijing 100191, China; 1911210153@pku.edu.cn (X.Y.); dyqhoney@126.com (Y.D.); 2 Department of Pediatrics, Peking University Third Hospital, Beijing 100191, China; yxsxz@outlook.com (Y.X.); adelebjmu@126.com (H.L.); changyanmei1226@163.com (Y.C.); 13911809214@163.com (Y.Y.); 3 National Engineering Center of Dairy for Maternal and Child Health, Beijing Sanyuan Foods Co., Ltd., Beijing 100163, China; liubin@sanyuan.com.cn; 4 Cuiying Biomedical Research Center, Lanzhou University Second Hospital, Lanzhou 730030, China; ery_wangqery@lzu.edu.cn

**Keywords:** preterm, prebiotics, glycomacropeptide, metagenomics

## Abstract

Microbial colonization of very preterm (VPT) infants is detrimentally affected by the complex interplay of physiological, dietary, medical, and environmental factors. The aim of this study was to evaluate the effects of an infant formula containing the specific prebiotic mixture of scGOS/lcFOS (9:1) and glycomacropeptide (GMP) on the composition and function of VPT infants’ gut microbiota. Metagenomic analysis was performed on the gut microbiota of VPT infants sampled at four time points: 24 h before the trial and 7, 14, and 28 days after the trial. Functional profiling was aggregated into gut and brain modules (GBMs) and gut metabolic modules (GMMs) based on the Kyoto Encyclopedia of Genes and Genomes (KEGG) pathways. *Enterococcus faecium*, *Escherichia coli*, *Klebsiella aerogenes,* and *Klebsiella pneumoniae* were dominant species in both the test group and the control group. After the 4-week intervention, the abundance of *Bifidobacterium* in the test group was significantly increased. We found two GBMs (quinolinic acid synthesis and kynurenine degradation) and four GMMs (glutamine degradation, glyoxylate bypass, dissimilatory nitrate reduction, and preparatory phase of glycolysis) were significantly enriched in the test group, respectively. The results of this study suggested that formula enriched with scGOS/lcFOS (9:1) and GPM is beneficial to the intestinal microecology of VPT infants.

## 1. Introduction

Globally, the preterm rate is rising and is the major cause of infant mortality. Preterm infants have immature and fragile organs, which leads them to be more susceptible to a series of health problems, especially when they are born very preterm (VPT) (born 28 to <32 weeks of gestation) [1]. Despite recent advances in neonatal care, VPT infants remain at high risk of necrotizing enterocolitis, respiratory problems, neonatal jaundice, and neurodevelopmental impairment [2].

Due to various health challenges, VPT infants are usually hospitalized in the neonatal intensive care unit (NICU) for an extended period of time, being put on artificial respiration and fed artificially or parenterally. Moreover, antibiotics and other medications are widely used during the treatment of these infants. All these factors may interfere with the natural pattern of microbiota acquisition and development, resulting in an aberrant establishment or deviation of the composition of the gut microbiota [3,4]. The microbiota of healthy term infants is dominated by *Bifidobacterium* and *Bacteroides*; however, these bacteria only exist in low abundance in premature infants [5]. In contrast, premature infants show low diversity and increased colonization of potentially pathogenic bacteria from Gram-negative *Enterobacteriaceae* of *Proteobacteria* [6]. These alterations may dramatically affect short- and long-term health [7].

Nutrition is a key factor in shaping the composition and function of the early microbiota [8]. Breastmilk is considered the gold standard of infant nutrition and is recommended for preterm infants [9]. However, preterm infants (especially <32 weeks of gestation) often face the dilemma of insufficient breast milk. In this condition, infant formula provides a healthy alternative that attempts to mimic the nutritional content of breast milk [10]. The most common infant formula is based on bovine milk, which has a very different nutritional composition than human breast milk. Human milk oligosaccharides (HMOs) not only have a “prebiotic” effect [11] but are also rich in sialic acid found in brain gangliosides [12], the content of which is significantly lower in formula than in breast milk [13]. Sialic acids are a class of alpha-keto acid sugars with a nine-carbon backbone [14]. A study on piglets confirmed that sialic acid derived from casein glycomacropeptide could improve the learning and memory abilities of piglets during early development [15].

Studies have found that the addition of prebiotics in term infant formula promotes the development of a neonatal gut microbiota resembling that of breast-fed infants [16]. Furthermore, feeding infant formula containing a GOS/FOS mixture has a positive effect on bifidobacterial abundance [17]. Glycomacropeptide (GMP) is a glycopeptide rich in sialic acid that can also promote the growth of beneficial bacteria and bind to pathogenic bacteria [18]. It has numerous biological effects on gut health, including preventing pathogen adhesion, decreasing intestinal barrier dysfunction, limiting lipopolysaccharide production, and attenuating inflammation [19].

However, most of the previous studies only focused on individual prebiotic-associated microorganisms and did not elucidate the whole gut microbiota of subjects; some studies reported gut microbial composition at the genus level without a detailed description of the species-specific effects of prebiotics. In the present study, we hypothesized that preterm formula supplemented with a prebiotic mixture of scGOS/lcFOS and GMP would facilitate the establishment of normal gut microbiota in VPT infants and confer health benefits. The aim of our study was to investigate the effects of a preterm infant formula enriched with scGOS/lcFOS and GMP on the composition and function of healthy VPT infants’ gut microbiota, using metagenomic data collected at four time points. The effects on infant growth and stool characteristics were further aims of this study.

## 2. Materials and Methods

### 2.1. Study Design and Subjects

This study is a prospective, non-randomized, controlled trial conducted between October 2019 and November 2020 in the NICU of Peking University Third Hospital. The study was approved by the Peking University Third Hospital Medical Science Research Ethics Committee (No. S2016159). It was registered in the Chinese Clinical Trials Register (registration number ChiCTR2100051988). Written informed consent was obtained from parents before infant enrollment in the study.

Healthy VPT infants born at a gestational age between 28 and 32 weeks and whose mothers could not offer sufficient breastmilk or elected not to breast-feed were recruited as the study subjects. Exclusion criteria included severe gastrointestinal dysfunction, congenital malformations, genetic diseases, or any other disease requiring surgery. The infants were assigned to receive either a standard formula (control group) or an experimental formula (test group) according to the preference of parents.

Basic clinical information about the parents and their offspring was obtained through the medical records, including the mother’s age, mode of delivery, gestational age, infant sex, Apgar score, antenatal and postpartum antibiotic therapy, birth weight, length, and head circumference.

Due to considerations of feeding tolerance and safety, the trial was started when the infants’ enteral feeding volume reached 80 mL/kg/d. Prior to this, both groups were fed the same standard formula for preterm infants. When the prescribed enteral feeding amount was reached, the test group was fed the experimental formula, and the control group continued to feed the standard formula. The control and experimental formulas were comparable in nutritional composition, except that the experimental formula contained a prebiotic mixture providing 0.65 g scGOS/lcFOS (9:1) and casein GMP providing 40 mg sialic acid/100 mL. Both formulas were bovine milk-based, containing energy 316 kJ/100 mL, protein 0.68 g/100 kJ, and lipid 1.30 g/100 kJ. The intervention was carried out when the infant was hospitalized in the NICU and lasted for 28 days. During this period, they underwent a daily clinical follow-up, including feeding volume, stool consistency, and stool frequency.

### 2.2. Stool Collection

Fresh stools were collected by a trained nurse from the infants’ diapers within 24 h before, 7 days after, 14 days after, and 28 days after the intervention using a collection tube containing 8 mL of DNA stabilizer reagent (PSP^®^ Spin Stool DNA Plus Kit, STRATEC Biomedical AG, Birkenfeld, Germany). The samples were kept frozen at −80 °C until DNA extraction.

### 2.3. Fecal DNA Extraction and Metagenomic Sequencing

The metagenomic DNA was extracted using MagPure Stool DNA kf kit (MP, Guangzhou, China), following the manufacturer’s instructions, which included a step of mechanical cell disruption by bead beating.

Metagenomic sequencing was performed by China National GeneBank (Shenzhen, China), following the protocol published previously [20]. Briefly, DNA was fragmented and barcoded, then subjected to amplification to produce DNA nanoballs. High-throughput sequencing was performed on BGISEQ-500. Adaptor and low-quality reads were removed, and human DNA reads were filtered out.

### 2.4. Taxonomic and Functional Profiling

MetaPhlAn 3.0 was used to estimate the relative abundance of taxonomic profiles. Putative amino acid sequences were translated from the gene catalog [21] and aligned against the proteins or domains in the Kyoto Encyclopedia of Genes and Genomes (KEGG) databases (release 79.0, with animal and plant genes removed) using BLASTP (v2.26, default parameter, except -m 8 -e 1e-5 -F -a 6 -b 50). Each protein was assigned to a KEGG orthologous (KO) group on the basis of the highest-scoring annotated hit(s) containing at least one segment pair scoring over 60 bits. The relative abundance profile of KOs was determined by summing the relative abundance of genes from each KO using the mapped reads per sample [21]. The abundance of each gut metabolic module (GMM) (-a 2 -d GMM.v1.07.txt -s average) and gut neuroactive module (GBM) (default parameter) were calculated as shown in the former article [22,23].

### 2.5. Statistical Analysis

All statistical analyses were performed using R statistical software version 4.0.3. Baseline characteristics and clinical results of the study participants were presented as mean ± SD for continuous variables and frequencies for categorical variables.

Alpha-diversity (Shannon index) was calculated using the Vegan package and compared by using Wilcoxon rank-sum test. Phylogenetic measures of beta-diversity based on the genus level abundance profile were also calculated by using the Vegan package, and PCoA plot based on Bray–Curtis distances were performed using the ggplot2 package. The top two principal coordinates (PC1 and PC2, representing the maximum amount of variation present in the dataset) were compared in each group. In order to investigate the specific differences in the gut microbiome composition and function between the test and control groups, Wilcoxon rank-sum test was used, and *p*-values were corrected for multiple testing using the Benjamin and Hochberg method. In order to compare the microbiota community structure across four time points, Kruskal–Wallis test was used and pairwise test for multiple comparisons was performed using Bonferroni’s *p*-adjustment method. Statistical significance was assumed at *p* < 0.05.

### 2.6. Data Availability

The datasets used to analyze for this study can be found in the China National Genebank (CNGB) with program ID CNP0001742.

## 3. Results

**Figure 1:**
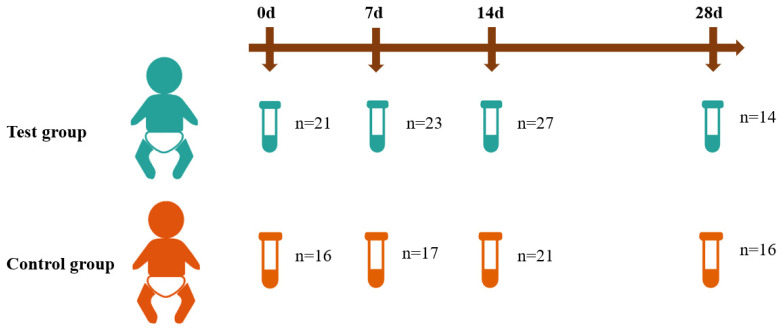
The study cohort. A total of 72 very preterm infants were included in this study, among which 37 infants were given a test formula containing prebiotics and sialic acid. Fecal samples were collected 24 h before, 7 days after, 14 days after, and 28 days after the intervention.

A total of 155 samples from day 0 (24 h before intervention), 7 days, 14 days, and 28 days after intervention were obtained from 72 infants (Figure 1). One participant in the test group dropped out because of sepsis, and one in the control group dropped out because of necrotizing enterocolitis, and they were not included in the analysis. In the test group, an average of 2.3 stool samples were collected per participant, and in the control group, an average of 2 stool samples were collected per participant. The characteristics of the participants are shown in Table 1. Thirty-seven infants were allocated to the test group and thirty-five to the control group in the present research. No significant differences were found in baseline characteristics between the two study groups except that the test group was more mature (*p* = 0.040) and had a shorter hospitality stay than the control group (*p* = 0.039). However, there was no significant difference in the corrected gestational age at the beginning of the intervention.

**Figure 2:**
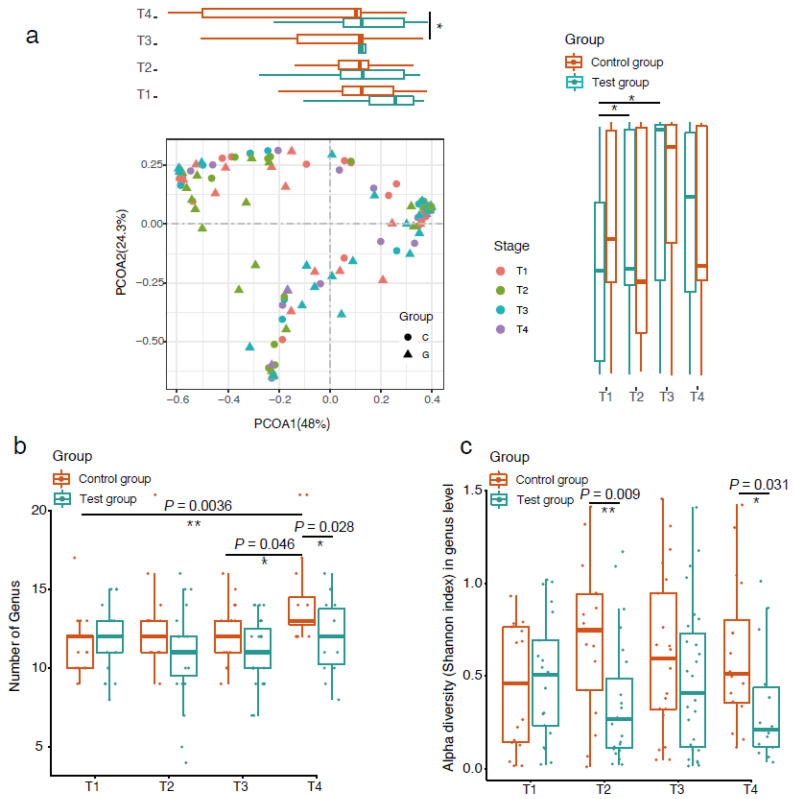
The effect of formula supplemented with prebiotics and sialic acid on microbial diversity in the VPT infant gut. PCoA of Bray–Curtis distances based on the profile of genera, * indicates difference at *p* value < 0.05, ** indicates difference at *p* value < 0.01 (**a**); comparison of genus numbers between the test group and control group (**b**); alpha diversity of gut microbiota measured by the Shannon index (**c**).

PCoA based on Bray–Curtis dissimilarity was used to examine the microbial community structure across different groups and stages (Figure 2a). The distribution of samples by groups and stages is shown along the first and second axes of the PCoA plot. Along the first axis, the value of PC1 in the control group showed an increasing trend over time, and there was a significant difference in microbiota community structure between stages three and four. A similar trend was found in the test group, but no significant difference was observed between the four stages. Along the second axis, the microbiota community structure of the test group showed significant differences between stages one and two, as well as stages one and three. There was no such difference found in the control group.

We compared the number of genera between the test group and the control group over the sampled time points (Figure 2b). We found that the genus number of the control group showed an increasing trend over time while the test group remained constant. The number of genera in the control group at stage four was significantly higher than that at stage one (*p* = 0.0036) and stage three (*p* = 0.046). After 28 days of intervention, the genus number of the control group was significantly higher than that of the test group, indicating that the test formula had an effect on the richness of microbiota in the VPT infant gut (*p* = 0.028).

The alpha diversity of the infant gut microbiota was measured by the Shannon index. There was no significant change in the microbial diversity within either group over the intervention period. When comparing between feeding groups, the test group was significantly lower than the control group at 7 d (*p* = 0.009) and 28 d (*p* = 0.031) (Figure 2c). 

**Figure 3:**
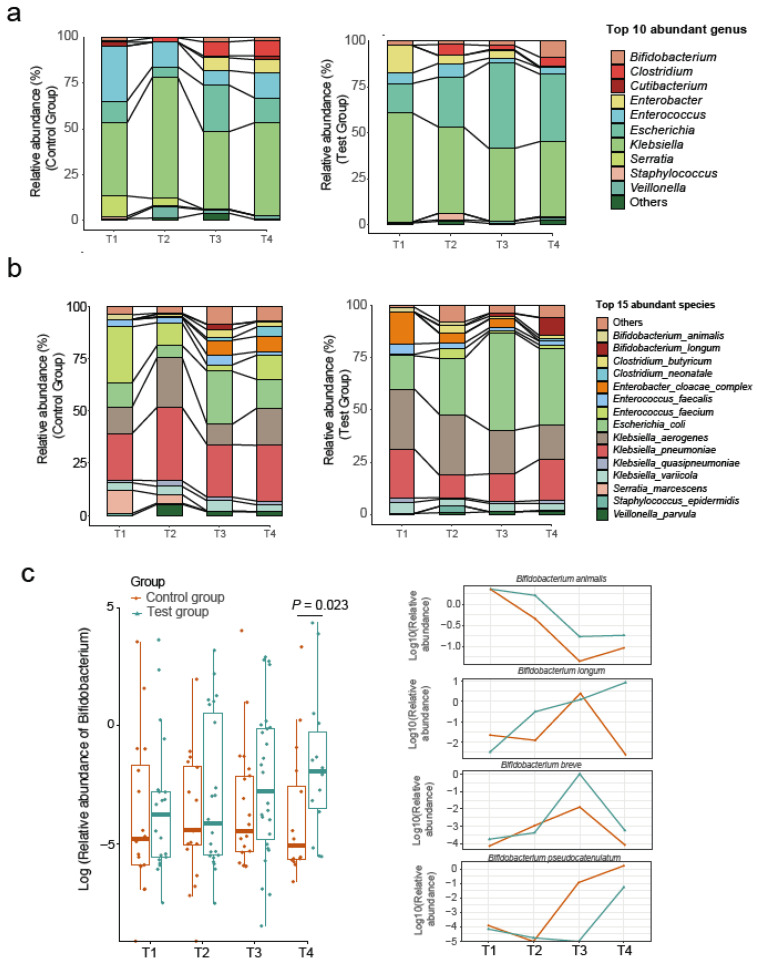
Relative abundance of gut microbiota at the genus level (**a**) and species level (**b**). Comparison of the relative abundance of *Bifidobacterium* between the test group and the control group (**c**).

The main phyla in the feces in both the test group and the control group were Proteobacteria, followed by Firmicutes and Actinobacteria. Consistent with the results of previous studies, we detected that *Enterobacteriaceae*-related genera such as *Klebsiella*, *Escherichia,* and *Enterobacter,* and genera of *Enterococcus*, *Clostridium,* and *Bifidobacterium* predominated the intestinal microbiome of VPT infants (Figure 3a). The relative abundance of the gut microbiota in both the test and control groups fluctuated during the intervention period.

We found that *Enterococcus faecium*, *Escherichia coli*, *Klebsiella aerogenes,* and *Klebsiella pneumoniae* were dominant species in both groups (Figure 3b). The test group was characterized by a higher relative abundance of *Bifidobacterium* on day 28 (adjusted *p* = 0.023, Figure 3c). Four *Bifidobacterium* species were detected: *B. longum*, *B. animalis*, *B. breve,* and *B. pseudocatenulatum*. Among them, *B. longum* was most affected by the administered prebiotics and showed the greatest increase in the test group (Figure 3c).

**Figure 4:**
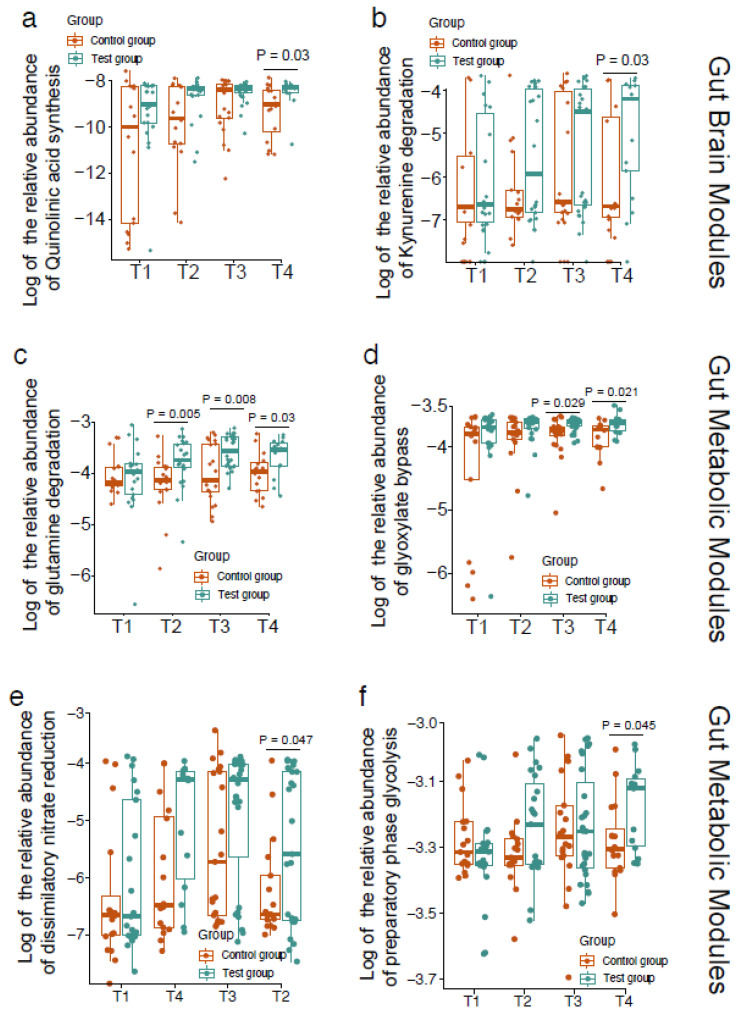
Comparison of gut and brain modules (GBMs) and gut metabolic modules (GMMs) between the test group and the control group. Quinolinic acid synthesis (**a**), kynurenine degradation (**b**), glutamine degradation (**c**), glyoxylate bypass (**d**), dissimilatory nitrate reduction (**e**) and preparatory phase of glycolysis (**f**).

Using gut and brain modules (GBMs) and gut metabolic modules (GMMs), we evaluated the functional capacity development of gut microbiota. After 28 days of intervention, two significantly changed GBMs between two groups were observed. Quinolinic acid synthesis and kynurenine degradation were enriched in the test group (Figure 4a,b). Four significantly changed GMMs between the two groups were observed. Glutamine degradation, glyoxylate bypass, dissimilatory nitrate reduction, and preparatory phase of glycolysis (Figure 4c–f) were enriched in the test group compared with the control group.

Infants in both groups consumed an increasing amount of formula during the study period. No significant difference was observed between the two groups in daily intake of formula, weight, length, and head circumference. No significant differences were shown at any timepoint for stool consistency and stool frequency between the test and control groups (Table 2). 

## 4. Discussion

To the authors’ knowledge, the present study was the first study using metagenomic shotgun sequencing to profile the gastrointestinal microbiome of VPT infants. We found that after the 4-week intervention of the specific prebiotic mixture of scGOS/lcFOS (9:1) and GPM, the abundance of *Bifidobacterium* in the test group was significantly increased. Gut and brain modules (GBMs) and gut metabolic modules (GMMs) showed differences in neuroactive compound production and energy source utilization between the test and control groups.

It is acknowledged that gestational age is an important factor in the establishment of the infant gut microbiota [24]. VPT infants have a delayed progression to a *Bifidobacterium*-dominated microbiota compared to term infants [6]. Consistent with previous studies [25,26], we observed that *Klebsiella*, *Escherichia,* and *Enterococcus* predominated the intestinal microbiome of VPT infants. Although the gut microbiota of VPT infants is highly dynamic, the composition and longitudinal progression trend of the gut microbiota in the two groups were similar because they were both fed with formula.

In our study, we found a significant increase in the abundance of *Bifidobacterium* in the test group, with a proportion similar to that reported in breast-fed counterparts [27]. Both scGOS/lcFOS and GPM have been reported to promote the growth of *Bifidobacterium*. A dose-related bifidogenic effect of GOS/FOS supplementation has previously been shown in formula-fed term infants, ranging between 0.4–0.8 g/dL [28], which was comparable to our study. On the other hand, Korpela et al. studied probiotic and galactooligosaccharides (GOS) supplementation in breast-fed and formula-fed neonates; they found the bifidobacterial community in the formula-fed infants have a weaker response to the supplement compared to the breast-fed infants. Only breast-fed infants showed the expected increase in bifidobacteria and reduction in *Proteobacteria* and *Clostridia* [29]. GMP supplementation promoted the growth of *Bifidobacterium* in the feces of rats with atopic dermatitis and increased the contents of acetic acid and butyric acid [30]. The administration of GMP to 6-week-old infants for 6 months augmented the levels of *Bifidobacterium* compared with baseline [31]. At the species level, we found 4 *Bifidobacterium* species in the VPT infant gut: *B. longum*, *B. animalis*, *B. breve,* and *B. pseudocatenulatum*. The former two were relatively abundant in the VPT infant gut, and *B. longum* showed the greatest response to the intervention. This finding was in correspondence with a previous study in bacterial cultures, which showed that *B.* *longum* strains had the ability to utilize both GOS and FOS and used a major extent for growth [32].

Moreover, the premature gut microbiota is different not only in composition but also in functionality. The main short-chain fatty acids (SCFAs) produced by the intestinal microbiota were found at lower levels in fecal samples from preterm and VLBW infants than in the feces of full-term infants [33]. In this study, we found that the intervention also improved the function of the microbial community. Glutamine is the amino group donor for many cellular biosynthetic reactions and serves as a storage reservoir of ammonia, playing a central role in nitrogen metabolism [34]. The glutamine degradation enriched in the test group might indicate the enhanced ability of microorganisms to utilize nitrogen sources. Glyoxylate bypass is also known as the glyoxylate shunt, in which acetyl-CoA is converted to succinate for the synthesis of carbohydrates. The glyoxylate shunt acts as a microbial survival pathway [35,36] and is essential for the production of bacterial acetate and fatty acid metabolism [37]. Dissimilatory nitrate reduction is a pathway related to bacteria’s respiration. Nitrate is one of the alternative electron acceptors allowing bacteria to respire in the absence of oxygen [38]. The glycolytic pathway is a major metabolic pathway for microbial fermentation, and the preparatory phase forms a key intermediate of the pathway. In the preparatory phase of glycolysis, one glucose molecule is converted to two glyceraldehyde-3- phosphate [39]. The kynurenine pathway is responsible for 90% of tryptophan metabolism, and the downstream metabolites kynurenic acid and quinolinic acid of this pathway have recently been identified as relevant for the nervous system, as they exert neuroprotective and excitotoxic effects, respectively, through their interaction with N-methyl-D-aspartate (NMDA) receptors [40]. Recently, it became evident that intestinal bacteria can affect brain function and behavior through signaling pathways of the microbiome gut–brain axis [41].

Since previous studies have reported that the preterm infant gut cluster is independent of sex or delivery mode [42], we did not subgroup these factors in the subject recruitment and analysis. Antibiotics are another factor that has an important influence on the intestinal microbiota. In this study, all infants received one course of antibiotics, with a third receiving at least one additional course. Thus, despite the potential confounding effects of antibiotic use, this study represents a typical characterization of the bacterial community of VPT infants under clinical supervision.

The present study has several limitations. Firstly, although the study was conducted out of humanitarian interest and left to parents to decide which preterm formula to feed their infants, this non-randomized study design may give rise to imbalances and biased estimates of treatment effects [43]. A relatively small sample size may limit the power of statistical analysis. However, our study found some significant differences in the composition and function of the gut microbiota between the test and control groups, which were consonant with previous findings. The loss of our study samples was mainly attributed to (i) extracting DNA from VPT infant stool samples being challenging and (ii) subjects being unable to adhere to our longitudinal intervention and collection schedule when discharged from the NICU. It is of great importance that safety is taken into account when trials are performed in VPT infants because those infants are at increased risk for infections, so the trials are conducted under close supervision in the NICU. This also resulted in a short trial period, which was insufficient to observe the trajectory trends of the intestinal microbiome over a long period of time.

## 5. Conclusions

In summary, our results show that formula enriched with scGOS/lcFOS (9:1) and GPM is safe for VPT infants, and it promotes the growth of *Bifidobacterium*. VPT infants fed the experimental formula have a microbiota more active in neuroactive compound production and energy source utilization, which might benefit their health. However, future randomized controlled studies with larger samples are warranted to further confirm these findings.

## Figures and Tables

**Table 1:** Characteristics of the study participants.

	Test Group (*n* = 37)	Control Group (*n* = 35)	*p*
Male sex (*n* [%])	14 (37.8)	20 (58.8)	0.077
Siblings at birth (*n* [% yes])	11 (29.7)	13 (37.1)	0.505
Cesarean delivery (*n* [%])	23 (62.2)	22 (64.7)	0.824
Maternal antibiotics at delivery (*n* [% yes])	11 (29.7)	10 (30.3)	0.958
Infant antibiotics after inclusion (*n* [% yes])	12 (32.4)	14 (40.0)	0.504
Maternal age (year)	32.2 ± 4.8	32.8 ± 4.7	0.589
Birth weight (g)	1329.2 ± 268.1	1265.1 ± 257.1	0.305
Birth length (cm)	38.3 ± 3.3	37.7 ± 2.5	0.384
Head circumference (cm)	27.6 ± 1.7	26.9 ± 1.7	0.060
1-min Apgar score	8.6 ± 2.0	8.2 ± 2.1	0.356
5-min Apgar score	9.4 ± 1.1	9.0 ± 1.2	0.227
10-min Apgar score	9.5 ± 0.8	9.4 ± 0.8	0.562
Gestational age at birth (week)	30.4 ± 1.8	29.5 ± 1.6	0.040
Corrected age at the beginning of intervention (week)	32.4 ± 1.3	31.8 ± 1.5	0.095
Hospitalization (day)	41.4 ± 15.2	49.4 ± 16.9	0.039

Continuous variables are represented as the mean ± SD.

**Table 2:** Clinical results at 4 time points.

	T1	T2	T3	T4
**Infant feeding volume (mL/day)**				
Test	175.1 ± 63.5	214.7 ± 69.5	258.4 ± 55.1	297.5 ± 38.0
Control	171.9 ± 78.3	199.0 ± 64.2	249.7 ± 59.7	293.9 ± 36.8
***p* value**	0.136	0.719	0.217	0.314
**Weight (g)**				
Test	1420.9 ± 199.9	1459.2 ± 186.4	1634.6 ± 199.5	1900.0 ± 197.2
Control	1393.1 ± 210.1	1456.8 ± 222.8	1599.1 ± 235.3	1900.0 ± 129.2
**p** **value**	0.582	0.966	0.583	0.699
**Length (cm)**				
Test	39.8 ± 2.9	40.3 ± 2.1	41.3 ± 2.3	44.7 ± 2.5
Control	38.8 ± 2.7	40.0 ± 2.6	41.1 ± 2.7	44.4 ± 2.8
***p* value**	0.231	0.736	0.814	0.794
**Head circumference (cm)**				
Test	28.0 ± 2.0	28.3 ± 2.0	29.1 ± 2.1	31.2 ± 3.1
Control	27.5 ± 1.8	28.2 ± 2.3	28.6 ± 2.3	29.7 ± 2.5
***p* value**	0.323	0.861	0.488	0.187
**Stool consistency (Bristol stool score)**				
Test	3.9 ± 0.5	4.0 ± 0.0	3.9 ± 0.4	4.0 ± 0.0
Control	3.8 ± 0.6	3.9 ± 0.3	3.9 ± 0.3	3.9 ± 0.4
***p* value**	0.359	0.103	0.865	0.253
**Stool frequency (times/day)**				
Test	1.7 ± 1.5	1.7 ± 1.3	2.2 ± 1.5	2.3 ± 1.1
Control	1.9 ± 1.2	2.1 ± 1.4	2.2 ± 1.3	1.7 ± 1.3
***p* value**	0.643	0.268	0.908	0.778

## Data Availability

The datasets used to analyze for this study can be found in the China National Genebank (CNGB) with program ID CNP0001742.
